# Supplementary material for: Preparation of CH3NH3PbI3 thin films with tens of micrometer scale at high temperature
Source: Sci Rep. 2017 Aug 16;7:8458. doi: 10.1038/s41598-017-09109-0 (PMC5559451; doi:10.1038/s41598-017-09109-0)
Supplement: Supplementary file 1 — Supporting information [file 41598_2017_9109_MOESM1_ESM.pdf]

Supplemental material

Preparation of $CH_3NH_3PbI_3$ thin films with tens of micrometer scale at high temperature

Hao Zhang [1], Mian Tao[1], Baizhi Gao[1], Wei Chen[1], Qi Li [1], Qingyu Xu [1,2,*], and Shuai Dong [1,*]

1. School of Physics, Southeast University, Nanjing 211189, China

2. National Laboratory of Solid State Microstructures, Nanjing University, Nanjing 210093, China

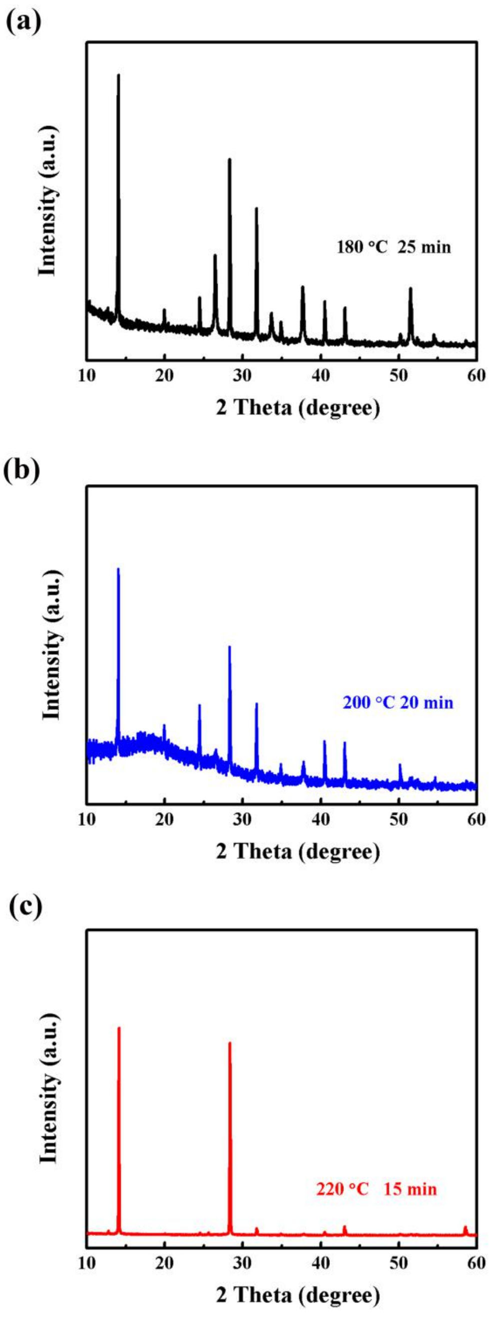

Figure S1 XRD pattern of $MAPbI_3$ thin films prepared (a) at 180 ^oC for 30 min, (b) at 200 ^oC for 20 min, (c) at 220 ^oC for 15 min.

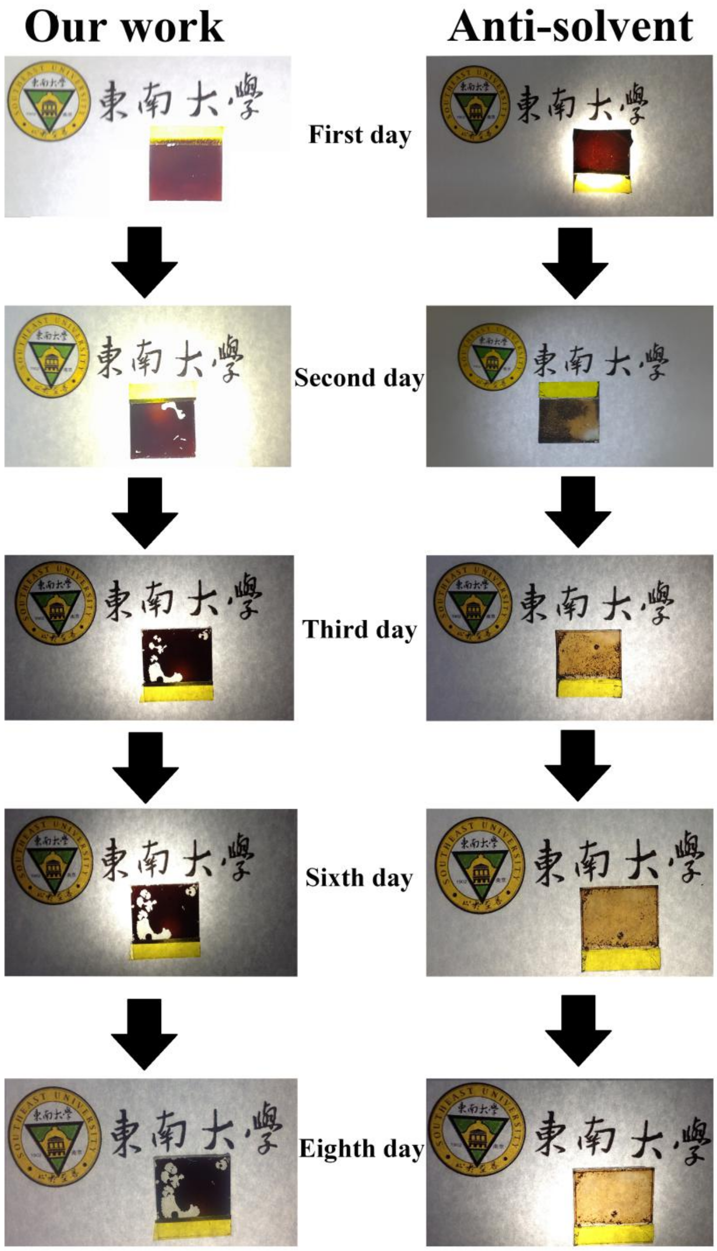

Figure S2 Photos of $MAPbI_3$ thin films prepared by our method and the anti-solvent method under the humidity of above 90% for various days.

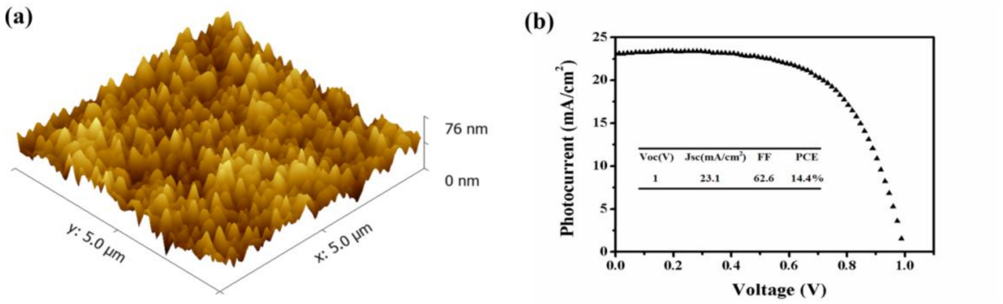

Figure S3 (a) Three-dimensional AFM image, the surface roughness (RMS value) is 10 nm and (b) J-V curve for the solar cell using $MAPbI_3$ film prepared by spin coating.

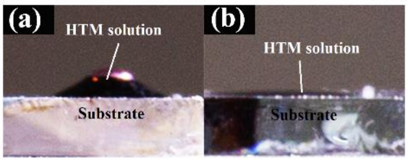

Figure S4 Images of contact angles between HTM chlorobenzene solution and $MAPbI_3$ thin films prepared by (a) our method and (b) the anti-solvent method.
